# Feasibility of Implementation of a Mobile Digital Personal Health Record to Coordinate Care for Children and Youth With Special Health Care Needs in Primary Care: Protocol for a Mixed Methods Study

**DOI:** 10.2196/46847

**Published:** 2023-09-20

**Authors:** David Y Ming, Willis Wong, Kelley A Jones, Richard C Antonelli, Nitin Gujral, Sarah Gonzales, Ursula Rogers, William Ratliff, Nirmish Shah, Heather A King

**Affiliations:** 1 Department of Pediatrics Duke University School of Medicine Durham, NC United States; 2 Department of Medicine Duke University School of Medicine Durham, NC United States; 3 Department of Population Health Sciences Duke University School of Medicine Durham, NC United States; 4 Department of Pediatrics Boston Children's Hospital Harvard School of Medicine Boston, MA United States; 5 Innovation and Digital Health Accelerator Boston Children's Hospital Boston, MA United States; 6 AI Health Duke University School of Medicine Durham, NC United States; 7 Duke Institute for Health Innovation Duke University School of Medicine Durham, NC United States; 8 Center of Innovation to Accelerate Discovery and Practice Transformation Durham Veteran Affairs Health Care System Durham, NC United States

**Keywords:** digital health, personal health record, children with special health care needs, care coordination, mixed methods, mobile phone

## Abstract

**Background:**

Electronic health record (EHR)–integrated digital personal health records (PHRs) via Fast Healthcare Interoperability Resources (FHIR) are promising digital health tools to support care coordination (CC) for children and youth with special health care needs but remain widely unadopted; as their adoption grows, mixed methods and implementation research could guide real-world implementation and evaluation.

**Objective:**

This study (1) evaluates the feasibility of an FHIR-enabled digital PHR app for CC for children and youth with special health care needs, (2) characterizes determinants of implementation, and (3) explores associations between adoption and patient- or family-reported outcomes.

**Methods:**

This nonrandomized, single-arm, prospective feasibility trial will test an FHIR-enabled digital PHR app’s use among families of children and youth with special health care needs in primary care settings. Key app features are FHIR-enabled access to structured data from the child’s medical record, families’ abilities to longitudinally track patient- or family-centered care goals, and sharing progress toward care goals with the child’s primary care provider via a clinician dashboard. We shall enroll 40 parents or caregivers of children and youth with special health care needs to use the app for 6 months. Inclusion criteria for children and youth with special health care needs are age 0-16 years; primary care at a participating site; complex needs benefiting from CC; high hospitalization risk in the next 6 months; English speaking; having requisite technology at home (internet access, Apple iOS mobile device); and an active web-based EHR patient portal account to which a parent or caregiver has full proxy access. Digital prescriptions will be used to disseminate study recruitment materials directly to eligible participants via their existing EHR patient portal accounts. We will apply an intervention mixed methods design to link quantitative and qualitative (semistructured interviews and family engagement panels with parents of children and youth with special health care needs) data and characterize implementation determinants. Two CC frameworks (Pediatric Care Coordination Framework; Patient-Centered Medical Home) and 2 evaluation frameworks (Consolidated Framework for Implementation Research; Technology Acceptance Model) provide theoretical foundations for this study.

**Results:**

Participant recruitment began in fall 2022, before which we identified >300 potentially eligible patients in EHR data. A family engagement panel in fall 2021 generated formative feedback from family partners. Integrated analysis of pretrial quantitative and qualitative data informed family-centered enhancements to study procedures.

**Conclusions:**

Our findings will inform how to integrate an FHIR-enabled digital PHR app for children and youth with special health care needs into clinical care. Mixed methods and implementation research will help strengthen implementation in diverse clinical settings. The study is positioned to advance knowledge of how to use digital health innovations for improving care and outcomes for children and youth with special health care needs and their families.

**Trial Registration:**

ClinicalTrials.gov NCT05513235; https://clinicaltrials.gov/study/NCT05513235

**International Registered Report Identifier (IRRID):**

DERR1-10.2196/46847

## Introduction

### Background

Care coordination (CC) is an essential intervention for children and youth with special health care needs that involves clinicians and patients or families collaborating to coordinate numerous health and related services across multiple service sectors within the “complex care ecosystem” of each child and youth with special health care needs [1]. Children and youth with special health care needs are children and youth aged 0-21 years that “have or are at increased risk for chronic physical, developmental, behavioral, or emotional conditions and who also require health and related services of a type or amount beyond that required by children generally” [2]. Among children and youth with special health care needs, family-centered CC is associated with better outcomes (eg, fewer emergency visits and missed school days) [1,3-6]; however, many children and youth with special health care needs receive inadequate CC [7,8] and 86% of children and youth with special health care needs lack a well-functioning system of services [2]. These critical CC gaps contribute to higher health care usage and parental stress and isolation [9] as parents and caregivers (referred to as families) of children and youth with special health care needs are forced to primarily manage their child’s CC needs in the absence of a well-functioning system of care [10].

To fill CC gaps, some families of children and youth with special health care needs have developed strategies to organize details of their child’s lifelong health records (eg, longitudinal care plans, manual notebooks, or binders) [11,12]; however, better solutions are needed because these approaches have limitations. For example, manually curated records by families are labor-intensive, not synchronized with electronic health records (EHRs), and largely invisible to clinicians, thereby limiting their impact and obscuring family insights into their child’s health progress at home. Digital health innovations offer promising solutions for children and youth with special health care needs and families to manage CC needs at home. In particular, digital personal health records (PHR) [13] enabled by Fast Healthcare Interoperability Resources (FHIR) for secure data access and sharing between mobile apps and EHRs [14,15] could assist families of children and youth with special health care needs to directly manage health information and facilitate family-centered CC [16]. FHIR-enabled digital PHRs accomplish this by synchronizing EHR and family-reported information with a personal mobile device, sharing family-reported insights longitudinally with providers (eg, care goals), and facilitating family and provider communication.

Despite their potential as digital health innovations and the clinical need for solutions for children and youth with special health care needs, FHIR-enabled digital PHRs have not yet been broadly adopted due to logistical and research challenges. Key logistical challenges include technical barriers, funding and infrastructure support, and lack of end user engagement in co-design [17]. In the research domain, as adoption of digital health innovations (eg, telehealth, mobile apps, and remote monitoring) accelerates, early phase pragmatic clinical research is needed [18] to elucidate implementation outcomes (eg, feasibility and acceptability) [19] and identify implementation barriers and facilitators that can inform future larger scale implementation efforts [20]. Methodologically, mixed methods research (MMR) is well-suited for pragmatic studies [21] but under-used in digital health research. MMR could be particularly valuable for digital health research because integration of multiple data sources can augment understanding of implementation more than quantitative or qualitative data alone [22,23]. As such, MMR provides an approach to achieve nuanced understanding of how, why, and for whom a digital health innovation might work best in real-world settings.

### Objectives

The objectives of this study are to (1) evaluate the feasibility of a digital PHR mobile app with FHIR-enabled EHR integration to coordinate care for children and youth with special health care needs, (2) identify and describe barriers and facilitators (determinants) of mobile app implementation, and (3) explore associations between mobile app adoption and patient or family-reported outcomes.

## Methods

### Study Design

**Figure 1:**
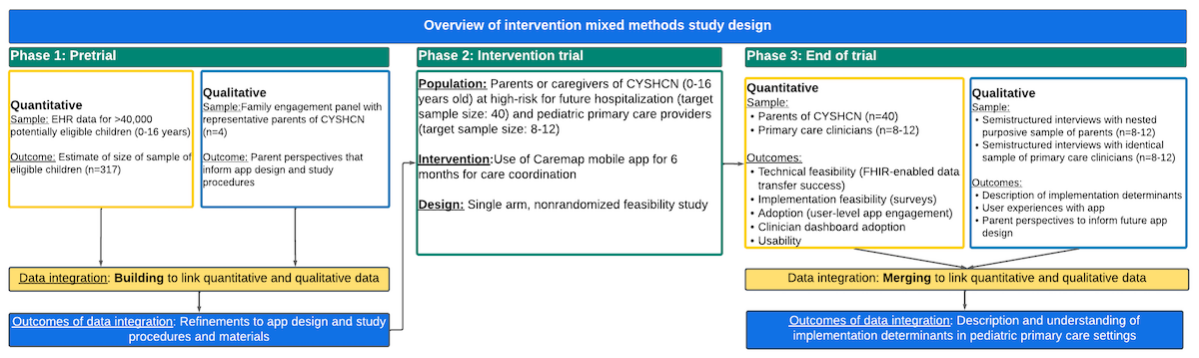
Overview of mixed methods study design and data integration method. CYSHCN: children and youth with special health care needs; EHR: electronic health record; FHIR: Fast Healthcare Interoperability Resources.

This study protocol describes a nonrandomized, single arm, prospective feasibility trial of a FHIR-enabled digital PHR mobile app used as a CC tool by families of children and youth with special health care needs in primary care settings for a 6-month period. We will apply an intervention MMR study design [24] within the clinical trial to link quantitative and qualitative data collection and analysis at multiple points in order to characterize implementation determinants (Figure 1).

### Intervention Description and Preliminary Proof-of-Concept Testing

**Figure 2:**
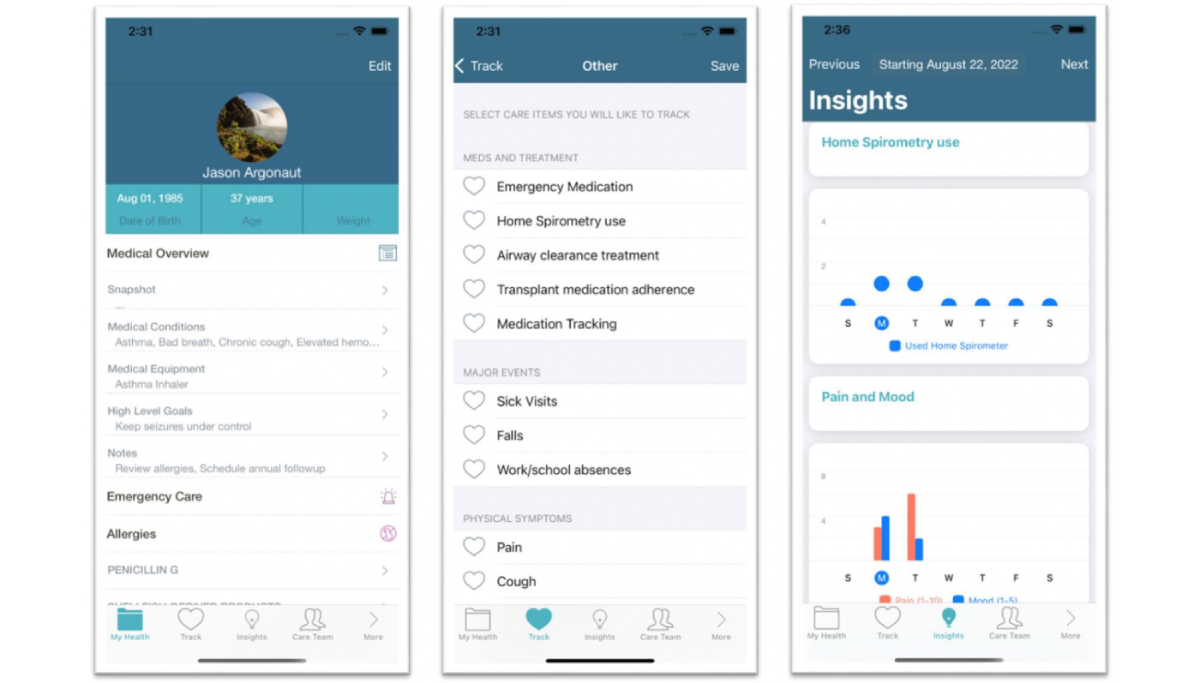
Caremap mobile app screenshots.

The intervention under evaluation in this study is a FHIR-enabled digital PHR mobile app (Caremap). The app is a digital health innovation designed to facilitate family-centered CC for children and youth with special health care needs through several key features. One key CC feature of the app is FHIR-enabled access to structured data elements from the child’s EHR chart (eg, medication list, allergies, and problem list), thereby allowing families to view health information as seen by their child’s primary care provider (PCP) in the EHR (Figure 2, left). A second key app feature is that families can track self-selected patient or family-centered care goals (eg, fewer missed school days due to illness and adherence to routine medications; Figure 2, middle) and graphically display progress over time (Figure 2, right) that is visible within the EHR to their child’s PCP via a clinician dashboard.

Core app features were developed to maintain the locus of control for the child’s health information with their families; development of the clinician dashboard involved input from practicing clinicians. After initial app development was completed, parents of children and youth with special health care needs with established pediatric specialty care within our health system and a sample of parents of children and youth with special health care needs with care external to our health system tested an early app prototype [25]. These early experiences of family users provided proof-of-concept for the app’s core features and laid the groundwork for this study protocol’s evaluation of the app in real-world settings.

### Study Setting and Population

This study will be conducted at a large health system in the southern United States that includes a tertiary care children’s hospital and multiple primary care pediatric practices using an enterprise-wide EHR system (Epic). The study will recruit adult parents or guardians of children and youth with special health care needs aged 0-16 years old with primary care established within a network of pediatric primary care sites that are academically affiliated with our institution.

PCPs will be recruited as study participants in order to include clinician perspectives on the feasibility of a dashboard as a companion to the mobile app. The clinician dashboard is accessible from within the EHR and includes a summary of information recorded by families on their personal device and synchronized with the EHR, thereby providing PCPs with view-only access to family-reported longitudinal health insights at the point-of-care during clinical visits.

### Eligibility Criteria

Eligibility criteria for study participation will include (1) patient age of 0-16 years, (2) primary care attribution to a participating site, (3) PCP clinical determination that the child has complex needs that can benefit from additional CC support, (4) child is at high-risk for hospitalization in the next 6 months defined using a novel EHR-based machine learning clinical predictive model [26], (5) English-speaking, (6) family has requisite technology at home (internet access and Apple iOS mobile device), and (7) an active web-based EHR (Epic) patient portal account to which parent or caregiver has full proxy access. An active web-based EHR portal account with parental proxy access is required at our institution for FHIR-enabled data access and sharing between the app, the child’s EHR chart, and the clinician dashboard within the EHR.

### Recruitment Procedures

**Figure 3:**
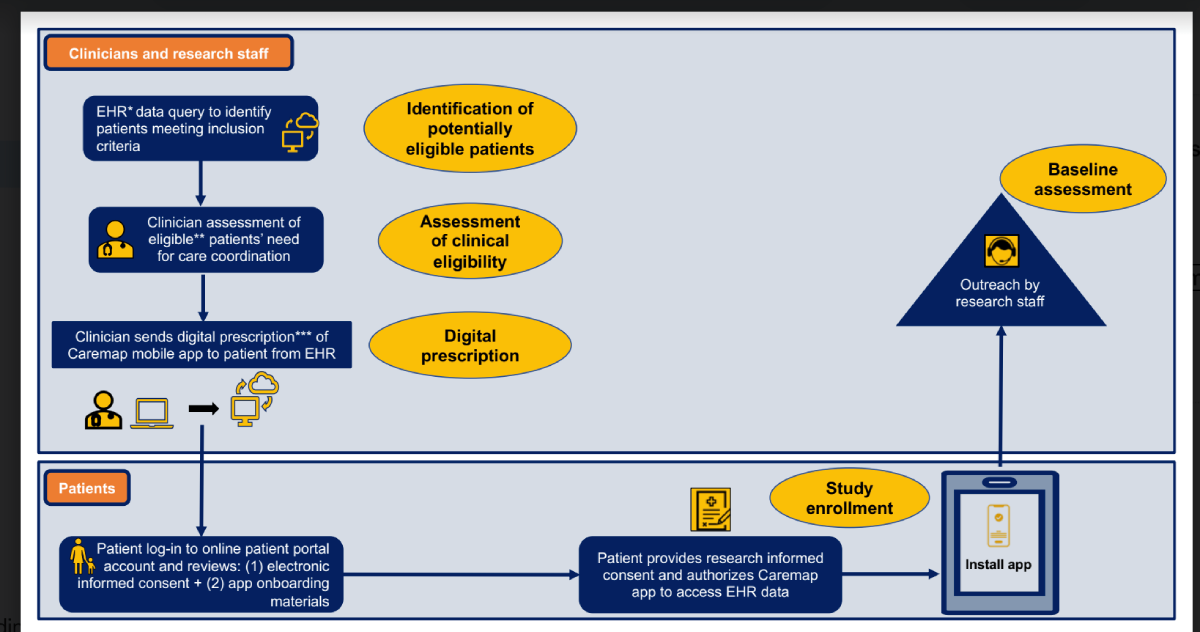
Study recruitment procedures. *EHR: electronic health records. **Eligible: established at pilot site + complex needs + active EHR online patient portal account (Epic MyChart©). ***Digital prescription: study materials and app onboarding materials sent via online patient portal to patient device.

A 3-step process (Figure 3) will be used to recruit and enroll the planned sample of 40 study participants. The first step—identification of potentially eligible patients—will be an EHR data query to identify patients meeting inclusion criteria identifiable within health system data (ie, 0-16 years old, primary care attribution to a participating site, active web-based portal account with full proxy access, English-speaking, and high-risk for future hospitalization).

The second step in recruitment—assessment of clinical eligibility—will be an assessment by each potentially eligible child’s PCP to determine whether the child has complex needs that can benefit from additional CC support. If the PCP determines that more CC support would be beneficial, the study will be introduced to parents of children and youth with special health care needs during an upcoming PCP office visit or via a mailed recruitment letter.

The third, final step of recruitment will be direct delivery of detailed study materials (eg, recruitment letter, study flyer, links for downloading the app, and tutorial videos and instructional guide for how to use the app) as a digital prescription (Figure 3). The digital prescription will be transmitted to parents of eligible participants via the patient’s web-based portal account. Transmission of digital prescription study materials will be facilitated by a digital health platform (Xealth) for prescribing and monitoring digital health tools within EHR workflows. After parents of children and youth with special health care needs review all materials, they will be able to consent or decline study participation from within the app (Apple Research Kit); those who consent will be contacted by research staff for baseline study assessment.

### Theoretical Frameworks

Guiding this study are 2 categories of extant theoretical frameworks focused on CC and evaluation (Table 1). The 2 CC frameworks central to this research are the Patient-Centered Medical Home (PCMH) and the Pediatric Care Coordination Framework [5,6]. The standard for longitudinal care of children and youth with special health care needs is the PCMH [27]; and CC is a core pillar of PCMH. The Pediatric Care Coordination Framework specifies functions and competencies for systems of care to meet patient or family needs across the ecosystem of essential services for children and youth with special health care needs (eg, medical, social, community, and schools).

To complement these 2 CC frameworks, in this study we are also applying 2 theoretical frameworks focused on evaluation: the Technology Acceptance Model (TAM) and the Consolidated Framework for Implementation Research (CFIR). The TAM is a theoretical model widely applied in digital health research to explain adoption and use of new technologies by end users [28]; the TAM proposes that technology use is mediated by users’ perceptions of usefulness and ease of use, leading to acceptance and actual use [29,30]. CFIR is an implementation framework [31,32] developed to comprehensively evaluate implementation barriers and facilitators (referred to as “determinants”) within and across multiple domains (process, individuals, intervention, inner setting, and outer setting) [33]. CFIR has been applied in combination with other frameworks [34] and across all phases of implementation research [35]. The combination of TAM and CFIR has been proposed to be complementary [36] and together are well-positioned to guide implementation of digital health innovations.

**Figure 4:**
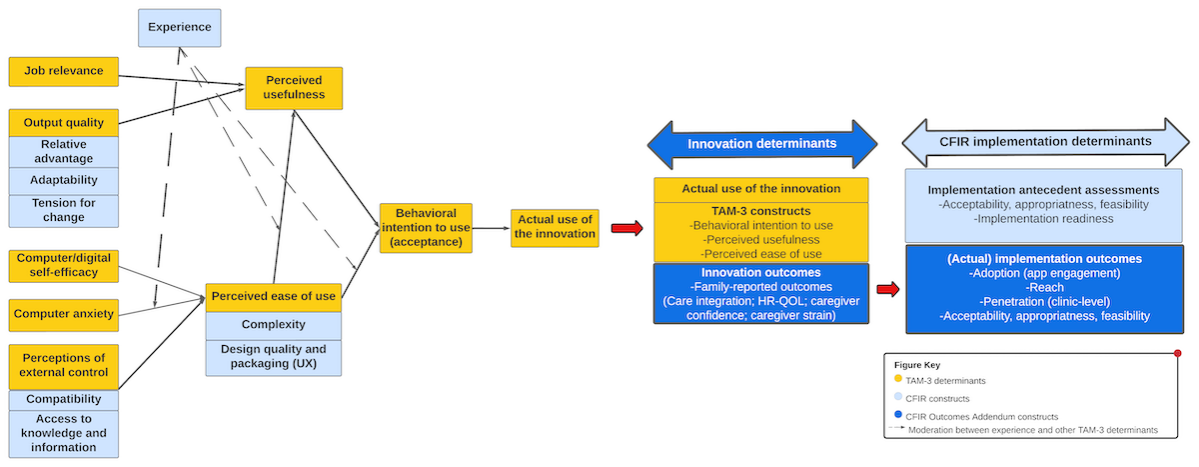
Applied conceptual framework: integration of CFIR, TAM-3, and CFIR Outcomes Addendum frameworks. CFIR: Consolidated Framework for Implementation Research. TAM-3: Technology Acceptance Model (version 3). CFIR Outcomes Addendum: adapted from from: Damschroder, et al [32] (CFIR), Venkatesh, et al [38] (TAM-3), and Damschroder, et al [40].

Despite potential for integration of CFIR with the TAM within digital health research [37], we are unaware of previously published examples. Our approach was to map core components of the Technology Acceptance Model (version 3; TAM-3) [38] and CFIR frameworks with one another to facilitate application of both in an integrated, parsimonious manner (Figure 4). An example of how these 2 theoretical frameworks can be integrated is that multiple CFIR-defined implementation determinants can be mapped to key TAM domains—for example, the CFIR-defined construct of compatibility aligns with perception of external control, which is a TAM-3-defined determinant of perceived ease of use [39]. The recently published CFIR Outcomes Addendum [40] provides additional theoretical foundations to inform this study’s conceptual framework. As defined by the CFIR Outcomes Addendum, the mobile app in this study is an example of an innovation and this study’s planned primary outcomes are “innovation outcomes.” These CFIR-defined innovation outcomes map to TAM-3–defined constructs (eg, behavioral intention to use, perceived usefulness, and perceived ease of use) that function as determinants of actual use of the innovation. Measurement of these innovation outcomes are then interrelated to CFIR implementation determinants and implementation outcomes (Figure 4).

**Figure 5:**
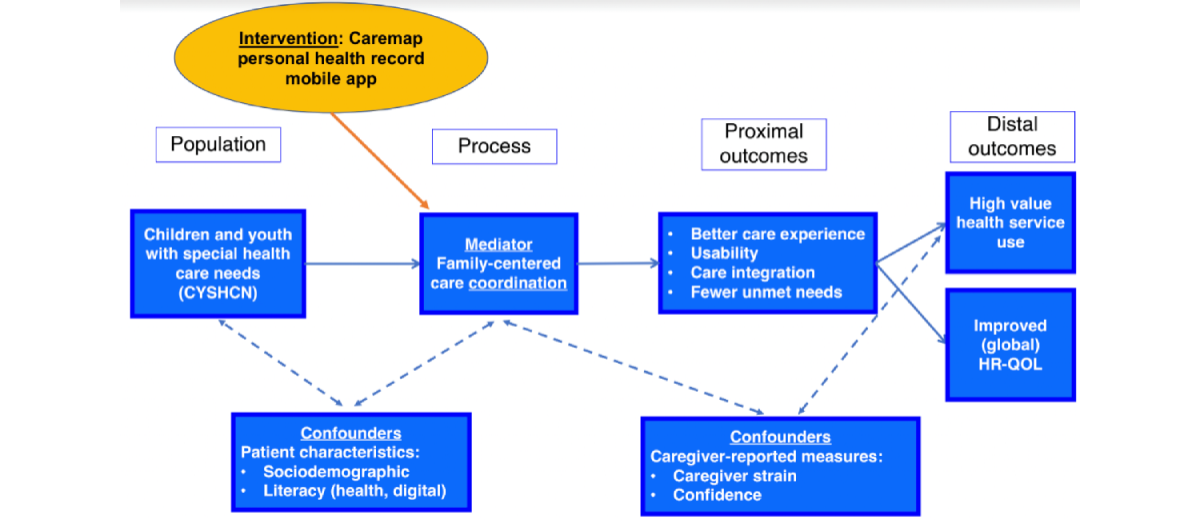
Study logic model. CYSHCN: children and youth with special health care needs; HR-QOL: health-related quality of life.

Selection of primary and secondary study outcome measures was guided by operationalizing the TAM-3 and CFIR theoretical frameworks as a study-specific logic model of hypothesized associations between mobile app use and process, proximal, and distal outcomes (Figure 5). Our study logic model was informed by a combination of published studies from the pediatric complex care and CC literature, our prior research, and clinical experience caring for children and youth with special health care needs.

**Table 1:** Overview of guiding theoretical frameworks.

Framework	Category	Purpose
Patient-Centered Medical Home	Care coordination	Gold-standard ambulatory care model with 5 core functions: accessible, comprehensive, patient or family-centered, coordinated, and high-quality or safe care
Pediatric Care Coordination Framework	Care coordination	American Academy of Pediatrics’ framework outlining purpose, scope, and features of care coordination
Technology Acceptance Model	Evaluation	Approach to understand reasons for acceptance and use of new technologies
Consolidated Framework for Implementation Research	Evaluation	Framework for assessing determinants (barriers and facilitators) of effective implementation of interventions

### Quantitative Measurement and Outcomes

A summary of quantitative measures is shown in Table 2. Feasibility is the primary outcome and will be measured as technical and implementation feasibility. Secondary outcome measures will be adoption (app log-ins by families and EHR dashboard use by clinicians) and parent-reported outcomes (eg, care integration and health-related quality of life) measured by parent-reported surveys.

**Table 2:** Summary of quantitative measures.

Measure name		Definition or approach	Frequency	Source
**Primary outcomes**				
	Technical feasibility	Measurement of key technical processes: (1) success rate of FHIR^a^-enabled data transfer from EHR^b^ to app and (2) success rate of FHIR-enabled transfer of parent-reported health insights from app to clinician dashboard	Weekly	Mobile app and EHR
	Implementation feasibility	Parent and clinician report of mobile app feasibility based on 4-item Feasibility of Intervention Measure	End of study (6 months)	Parent and provider surveys
**Secondary outcomes**				
	Adoption	Measurement of: (1) app engagement (number of log-ins), (2) clinician engagement (view of EHR clinician dashboard), and (3) number of times mobile app digitally prescribed to families by clinicians	Weekly (parents), monthly (clinician)	Mobile app and EHR
	Care integration	Parent report of degree of care integration using 19-item Pediatric Integrated Care Survey	Baseline, 6 months	Parent survey
	HR-QOL^c^	Parent report of HR-QOL for child using 9-item PROMIS^d^ global HR-QOL survey (parent-proxy)	Baseline, 3 months, 6 months	Parent survey
	App usability	Parent and clinician report of app usability based on 10-item System Usability Scale	Baseline, 6 months	Parent and clinician survey
	Caregiver strain	Parent report of degree of strain experienced by providing care for their child’s health conditions using the 7-item Caregiver Strain Questionnaire-Short Form 7	Baseline, 3 months, 6 months	Parent survey
	Parent confidence	Parent report of level of confidence in avoiding hospitalization using single item	Baseline, 3 months, 6 months	Parent survey
	Net promoter score	Parent report of likelihood of recommending the (Caremap) app to others using single item	End of study (6 months)	Parent survey
	Global health status	Parent-reported global assessment of child’s general health status	Weekly	Parent survey
	Implementation outcomes	Parent and clinician report of mobile app acceptability and appropriateness based on 4-item Acceptability of Intervention Measure and 4-item Intervention Appropriateness Measure	End of study (6 months)	Parent and provider surveys

^a^FHIR: Fast Healthcare Interoperability Resources.

^b^EHR: electronic health record.

^c^HR-QOL: health-related quality of life.

^d^PROMIS: Patient Reported Outcomes Measurement Information System.

### Qualitative Measurement and Outcomes

Qualitative measures, data collection, and analytical procedures are summarized in Table 3. The primary objective for qualitative data collection will be to identify and describe key implementation determinants (study aim 2). One source of qualitative data will be semistructured interviews with a nested purposive sample of 8-12 parents of children and youth with special health care needs and 8-12 PCPs participating in the study; we expect that this interview sample size will be sufficient to reach information saturation and information power [41,42]. The approach for participant interviews will be qualitative description [43]; interviews will be recorded and analyzed using rapid and value-adding qualitative analysis [44,45].

A second source of qualitative data will be family engagement panels (FEP) with a group of parents of children and youth with special health care needs purposively sampled to reflect a diversity of lived experiences. FEPs are modeled after Community Engagement Panels, a consultative model that engages patients, families, and other community stakeholders for project-specific input [46]. Rooted in principles of community-engaged research, these panels emphasize the importance of bidirectional communication, colearning, flattening of the hierarchy between researchers or project leaders and community stakeholders, and identifying mutual benefits of the project [47]. An initial FEP was conducted in fall 2021 prior to trial launch to gather formative feedback. A second FEP after completion of data analysis will be conducted to return findings to community partners and gather recommendations for future directions with an emphasis on enhancing inclusion and access with future app iterations.

**Table 3:** Summary of qualitative measures.

Qualitative data type	Approach	Timing	Analyses
Semistructured interviews (parents)	In-depth interviews with nested convenience sample of parents of children and youth with special health care needs (n=8-12) participating in the study; interview guide mapped to TAM^a^ and CFIR^b^ framework constructs	End of study	Rapid qualitative
Semistructured interviews (clinicians)	In-depth interviews with identical samples of primary care pediatricians (n=8-12) caring for study participants; interview guide mapped to TAM and CFIR framework constructs	End of study	Rapid qualitative
Family engagement panels	Small group discussions with purposive sample of 4-6 parents of children and youth with special health care needs to gather stakeholder input on study design or procedures and future direction	Baseline (prestudy), end of study	Qualitative description

^a^TAM: Technology Acceptance Model.

^b^CFIR: Consolidated Framework for Implementation Research.

### Integration of Quantitative and Qualitative Data

To address the study aim of identification and description of implementation determinants, we will use the MMR data integration method of embedding to link quantitative and qualitative data collection and analysis at multiple points within the intervention MMR study design [24] Consistent with an embedded data integration method, we will also apply other data linking strategies [24]. For example, we will use building to link pretrial FEP qualitative data with quantitative counts of eligible patients identified in EHR data to inform study recruitment procedures; and we will use merging to link collection and analysis of quantitative and qualitative data (feasibility data, surveys, and interviews) after the trial period. An overview of the intervention mixed method study design and embedded data integration methods is shown in Figure 1.

### Ethics Approval

The study protocol has been reviewed and approved by our institutional review board Duke Health (Pro00109514). Informed consent for eligible parents or caregivers will occur on their personal device. Eligible participants will review and sign the written electronic informed consent form prior to beginning the study. Participant confidentiality will be protected by collecting only information needed to assess study outcomes, and study data used for final summative quantitative analyses will be deidentified. All qualitative interviews will be conducted by a trained interviewer from the study team on the telephone, via video call (Zoom), or in-person. Interviews will be video-recorded or audio-recorded and transcribed; if an interviewee wishes to not be recorded, detailed notes will be taken instead. Parent or caregiver participants will receive US $50 compensation for completion of surveys (US $25 at baseline, US $25 at 6 months); participants who complete an interview will receive an additional US $50 compensation.

## Results

Participant recruitment began in October 2022. We expect that data analyses will be completed by early 2024. We anticipate that quantitative and qualitative findings will address primary and secondary outcomes outlined in Table 2.

Analyses of pretrial quantitative and qualitative data gathered to-date have informed study procedures and design. A pretrial FEP was conducted in fall 2021 with 4 parents of children and youth with special health care needs. Formative qualitative feedback gathered during this first FEP informed changes to app design and user-facing materials, monetary incentives for study participation, and recruitment procedures. An initial EHR data analysis was conducted in January 2022 to estimate the size of the eligible population for the study. From a population of >40,000 children with primary care attributed to the 3 participating PCP sites, our preliminary analysis identified 317 patients who met all inclusion criteria based on available EHR data; it is expected that this sample will be of sufficient size to enroll the goal of 40 study participants to use the mobile app.

We have used these preliminary quantitative and qualitative findings to refine study recruitment procedures. Specifically, based on feedback from parents of children and youth with special health care needs during the first FEP, recruitment procedures were revised to introduce the study to eligible patients directly during a PCP office visit instead of via automated web-based patient portal electronic messaging. Additionally, detailed patient-facing videos and written tutorials were developed in response to family requests for user-friendly onboarding materials. Quantitative estimate of the potentially eligible patient population led to procedural revisions so that PCPs will be familiar with study aims (to facilitate introduction of the study during office visits) and study materials can be delivered via digital prescription in order to better align with real-world clinical workflows.

## Discussion

### Anticipated Principal Findings

Findings from this study will characterize the feasibility and implementation barriers and facilitators (determinants) for a digital PHR mobile app used by parents of children and youth with special health care needs for CC in primary care settings. Novel study procedures that can contribute to the digital health literature include the protocol’s use of digital prescriptions for participant recruitment. Additionally, application of MMR and implementation research methods offers a novel methodological approach that could address recent calls for innovative and pragmatic evaluation of digital health solutions [48].

This study protocol describes a pragmatic approach for integrating theory into digital health study design by combining the established CFIR and TAM-3 frameworks [28,32,33,38], thereby facilitating application of implementation science methods within early phase clinical studies. It is recognized within the implementation research field that earlier phase research—for example, pilot and feasibility studies [20]—presents opportunities to establish foundations for future intervention dissemination and to test promising implementation strategies within the context of implementation determinants. Additionally, available implementation science models, theories, and frameworks [31] provide a strong theoretical basis for digital health intervention studies to integrate implementation science and digital health research frameworks.

Similar to implementation science, methodological approaches from the MMR field that systematically integrate quantitative and qualitative data [21,22] can generate insights needed to understand how best to implement, evaluate, and improve digital health innovations. In our preliminary work to-date, use of the MMR analytical technique of building to integrate pretrial quantitative and qualitative data informed improvements in study procedures that would have been challenging to identify without mixed methods data integration. We anticipate that continued collection of both forms of data with embedded data linking at multiple points in the trial will deepen understanding of the feasibility and implementation of the digital health mobile app. These methodological approaches are well-positioned for exploration and adoption by other digital health researchers.

In addition to integrating MMR and implementation science methods into digital health research, there are opportunities to streamline study procedures. For this protocol in particular, the use of digital prescriptions will be used to facilitate dissemination of study materials directly to eligible participants via existing web-based patient portal accounts. This approach may help improve study workflow efficiency and mitigate infectious risk in the face of the ongoing COVID-19 pandemic by reducing study personnel’s reliance on in-person recruitment. Digital prescriptions are also positioned for future studies where they could facilitate scaled recruitment via automated delivery of study materials to large numbers of eligible patients identified by EHR data. Although a digital prescription-based approach is promising for study recruitment, caution should be exercised. More efforts are needed to close the digital divide [49], mitigate inequitable access to web-based patient portals [50], and provide tailored support for historically marginalized populations (eg, instructions for web-based portal access and use in multiple languages; interpreter access for patients with limited English proficiency).

This study has several limitations that should be acknowledged, including the study’s single center design and limited size and scope. Smaller scope is necessary at this early stage to focus on feasibility and to gather in-depth feedback from participants that can inform subsequent app improvements. Additionally, our inclusion criteria that study participants have existing internet access, a digital device, and an active web-based patient portal account may introduce barriers to research participation. Factors that could mitigate these barriers include national-level data that demonstrated that 81% of US adults own a smartphone [51] and data from our previously conducted digital health trial when parents were successfully recruited using similar technology requirements [52]. Finally, this study’s lack of randomization and effectiveness evaluation will limit the strength of conclusions. Although evaluation of effectiveness of the mobile app on health outcomes is beyond the scope of this study, we will systematically explore family and clinician-reported data by including validated measures of health-related quality of life and perceptions of care integration [53,54].

Digital health tools have the potential to improve care and outcomes for children and youth with special health care needs; however, the evidence base is early in development. The methodological rigor of early phase digital health studies could be enhanced by incorporating novel study procedures and methods from research fields outside of traditional pediatric clinical trials. By doing so, strengthened conclusions from early phase digital health studies will be positioned to serve as foundations for future multisite prospective trials that measure effectiveness for health and implementation outcomes. Ultimately, these future directions can serve to accelerate the pace of investigation so that clinicians and families of children and youth with special health care needs will have the evidence base needed to determine the impact of new digital health tools on patient and family-centered outcomes.

### Conclusions

This study will evaluate the feasibility of integrating a FHIR-enabled digital PHR mobile app as a CC tool for children and youth with special health care needs into clinical care. Application of MMR and implementation science methods is anticipated to facilitate understanding of implementation determinants in primary care settings. The findings of this study are positioned to inform future pragmatic trials that can further advance knowledge of how to use digital health innovations for improving care and outcomes for children and youth with special health care needs.

## Appendix

Multimedia Appendix 1 Peer-review reports.

## Abbreviations

CC: care coordination
CFIR: Consolidated Framework for Implementation Research
EHR: electronic health record
FEP: family engagement panel
FHIR: Fast Healthcare Interoperability Resources
MMR: mixed methods research
PCMH: Patient-Centered Medical Home
PCP: primary care provider
PHR: personal health record
TAM: Technology Acceptance Model
TAM-3: Technology Acceptance Model (version 3)

## Data Availability

The data sets generated during or analyzed during this study are available from the corresponding author on reasonable request.
