# Supplementary material for: Feasibility of Implementation of a Mobile Digital Personal Health Record to Coordinate Care for Children and Youth With Special Health Care Needs in Primary Care: Protocol for a Mixed Methods Study
Source: JMIR Res Protoc. 2023 Sep 20;12:e46847. doi: 10.2196/46847 (PMC10551780; doi:10.2196/46847)
Supplement: Multimedia Appendix 1 [file resprot_v12i1e46847_app1.pdf]

SUMMARY STATEMENT
(Privileged Communication)

PROGRAM CONTACT:
Sheena Patel
sheena.patel@ahrq.hhs.gov

Release Date: 06/17/2021
Revised Date:

Application Number: **1 R21 HS028699-01**

Principal Investigator

MING, DAVID Y

Applicant Organization: DUKE UNIVERSITY

Review Group: HITR
Healthcare Information Technology Research

Meeting Date: 06/03/2021
Council: OCT 2021
Requested Start: 09/01/2021
RFA/PA: PA17-246
PCC: CEPI

Project Title: Feasibility Study of a Mobile Digital Personal Health Record for Family-Centered Care Coordination for Children and Youth with Special Healthcare Needs
SRG Action: Impact Score:29 Percentile:8 +

Human Subjects: 30-Human subjects involved - Certified, no SRG concerns
Animal Subjects: 10-No live vertebrate animals involved for competing appl.
Gender: 1A-Both genders, scientifically acceptable
Minority: 1A-Minorities and non-minorities, scientifically acceptable
Clinical Research - not NIH-defined Phase III Trial

Project Year	Direct Costs Requested	Estimated Total Cost
1	85,873	141,294
2	96,407	158,626
TOTAL	182,280	299,920

ADMINISTRATIVE BUDGET NOTE: The budget shown is the requested budget and has not been adjusted to reflect any recommendations made by reviewers. If an award is planned, the costs will be calculated by Institute grants management staff based on the recommendations outlined below in the COMMITTEE BUDGET RECOMMENDATIONS section.

RESUME AND SUMMARY OF DISCUSSION: This study R21 health services research grant application from Dr. David Ming, from Duke University, in Durham, NC, proposes a study to evaluate the feasibility of a digital personal health record (PHR) mobile application integrated with electronic health records by Fast Healthcare Interoperability Resources (FHIR) data standards to enhance care coordination for families of children and youth with special healthcare needs (CYSHCN). The reviewers agreed on the high significance of this proposed study noting that twenty percent of children have special health care needs and require care coordination between multiple subspecialists and communication of family health insights at home, and potentially care across disparate systems. They added that significant care coordination needs apply to adults with multiple care conditions (MCC) as well as children with special care needs, so success would potentially impact other patient groups. The reviewers also noted that the study is highly innovative. The proposed study will develop the first FHIR-enabled digital personal health record mobile app to improve care coordination with secure access and sharing of EHR data and family reported health insights, not yet been tested for regular clinical care. It includes a strong scientific premise, Good experimental design and evaluation. Good description of technology, bidirectional sharing of information, and a prototype that already exists. The research team is building on significant track record of work that logically leads to this study. However, the reviewers noted some minor weaknesses. The study should focus more on how the app will address underserved families from lower socio-economical backgrounds, who face literacy challenges, and who could particularly benefit from care coordination. The study relies on Fast Healthcare Interoperability Resources (FHIR) technology, which is not universally implemented and available, potentially limiting generalizability of the findings. The methods could better describe the details needed for the qualitative interviews and the degree to which intervention will fit into the clinical workflow. Also, the reviewers noted concerns with a limited sample size of families and providers involved. Based on the Scientific and Technical Merit of the application, this application received a score of 29.

DESCRIPTION (provided by applicant):
Coordinating care across a complex web of providers and services on their own is stressful and isolating for families of children and youth with special healthcare needs (CYSHCN); thus, better care coordination solutions are critically needed. Digital personal health records (PHR) that allow families to securely access, manage, and share their child's health data across multiple information and electronic health record (EHR) systems are promising solutions. Central to digital PHRs being able to fulfill their promise are Fast Healthcare Interoperability Resources (FHIR) – updated data standards that facilitate secure data access and sharing between third party mobile applications (apps) and EHRs. Despite their importance, FHIR technologies have yet to be widely adopted. A FHIR-enabled digital PHR mobile app (Caremap) was co-designed with families and clinicians to coordinate care for CYSHCN and has been positively reviewed by families in preliminary testing. Implementation and evaluation of the Caremap mobile app in real-world setting is now the critical next step forward. We will conduct a single group, non-randomized, feasibility study with 40 families (adult parents/caregivers) of CYSHCN in pediatric primary care clinics to use the digital PHR mobile application as a tool for coordinating their child's care over a 6-month period. The primary study outcome of overall feasibility will include measures of technical feasibility (FHIR-enabled integration of the mobile app with the EHR) and implementation outcomes in real-world clinical environments. Using a convergent mixed methods design, we will merge quantitative feasibility data and two qualitative data sources (semi-structured interviews with families and providers; family stakeholder engagement panels) to identify implementation barriers and facilitators. Using pre/post analyses of baseline and 6-month family-reported survey measures, we will explore associations between level of family app adoption and family-reported outcomes. This proposal is highly innovative because it will be the first FHIR-enabled digital PHR mobile app implemented to enhance care coordination for CYSHCN via secure access and sharing of EHR data and family-reported health insights. The findings will be highly significant because they will support a future multi-site pragmatic, cluster randomized trial to evaluate the effectiveness of enhanced care coordination

with a digital PHR mobile app compared to usual care coordination. Due to similarities in needs between CYSHCN and adults with multiple chronic conditions, the study's findings will inform improvements in family-centered care coordination that will be highly impactful for complex populations across the age spectrum.

PUBLIC HEALTH RELEVANCE: This study will evaluate the feasibility of a digital personal health record (PHR) mobile application integrated with electronic health records by Fast Healthcare Interoperability Resources (FHIR) data standards to enhance care coordination for families of children and youth with special healthcare needs (CYSHCN). The study will enroll 40 families (adult parents/caregivers) of CYSHCN in pediatric primary care clinics to use the digital PHR mobile application as a tool for coordinating their child's care over a 6-month period. Using a single group, non-randomized study design and convergent mixed methods analyses, the study will: (a) determine the feasibility of FHIR-enabled integration of the mobile application with electronic health records for care coordination; (b) identify barriers and facilitators to implementation in real-world settings; and (c) examine associations between level of app adoption by families and family-reported outcome measures.

CRITIQUE NOTE: The sections that follow are the essentially unedited, verbatim comments of the individual committee members assigned to review this application. The attached commentaries may not necessarily reflect the position of the reviewers at the close of group discussion, nor the final majority opinion of the group. The above RESUME/SUMMARY OF DISCUSSION represents the evaluation of the application by the entire committee.

CRITIQUE 1

Significance:	2
Investigator(s):	1
Innovation:	3
Approach:	4
Environment:	1

Overall Impact: CYSHCN is a very important subset of pediatrics patients group who need a majority of sources in pediatric care, but also suffer from care coordination problems. The proposed app has strong features such as utilizing SMART/FHIR functions to be embedded in EHR and improves family-care team interaction and information exchange. The proposal is well written with clear description of app, its features, and aims to be studied. The approach is well written with some minor issues to be easily fixed. It would be also important to consider Health Literacy level of users in their analysis for adoption, implementation as well as barriers to use the app.

1. Significance:
Strengths
- CYSHCN is an important subset of pediatrics patients group who need a majority of sources in pediatric care, but also suffer from care coordination problems.
- Families are safety net for the care of CYSHCN, and need support in the care of their kids.
- The proposed app has strong features such as utilizing SMART/FHIR functions to be embedded in EHR and improves family-care team interaction and information exchange.
- Very well written and conceived proposal.
- Pilot data shows prior acceptability, high usability of the product, in fact it is already offered in Apple app store for free download and use by any parents.

Weaknesses
- Underserved and under educated families are the ones who suffer most in the care coordination of their medically complex kids. The researchers should consider this, and make sure to recruit and show the outcomes from this group of families' perspectives.
- Health literacy should be collected and considered in the analysis.

2. Investigator(s):
Strengths
- Very strong team.

Weaknesses
- None noted.

3. Innovation:
Strengths
- Innovation comes with one of the first app to be used for the care coordination of complex care kids with EHR integration.

Weaknesses
- None noted.

4. Approach:
Strengths
- The approach is well written with all required steps to address each aims.
- Using the conceptual frameworks such as CFIR, TAM is a plus.
- Pre-Post design with a group of 40 parents.
- Detailed description of the app, how it works, and all limitations with alternative plans proposed.

Weaknesses
- We are not sure if 40 would be sufficient sample size especially with the quantitative survey analysis.
- Some of the sample sizes are not clear. For instance, they need to clarify that if 10-12 parents for the interview part (barriers and facilitators) are among those 40 parents, and how they pick them. Because there is one cohort group, I would expect to see they interview at least half of the sample to capture barriers and facilitators, since there might be significant difference in type of patient diseases, health literacy level of parents, parents' experiences due to severity or needs of their children's situation.
- In aim 2, They only interview 4 providers, which is really small sample size. This app is an interactive app, so providers' involvement and engagement is necessary to make this app successful for parents. That being said, they should interview at least 10 providers to get meaningful result from providers' perspective.
- The PI talks about second source of qualitative data source as the "family panel", however do not provide any more information. Who are these family panel? Are they among those 40 parents? If they are not, are they potential users? How do you make sure their perspectives (if not users) will not bias the data you get from your primary sample of 40 users (intervention group?)

5. Environment:
Strengths
- Great environment with all needed support.

Weaknesses
- None noted.

Appendix: Acceptable.

Budget and Period of Support: Acceptable.

Protections for Human Subjects: Acceptable.

Data and Safety Monitoring Plan: Acceptable.

Single IRB for Cooperative Research: Acceptable.

Inclusion of Women: Acceptable.

Inclusion of Minorities: Acceptable.

Inclusion of Individuals Across the Lifespan:
Acceptable.

Inclusion of Priority Populations:
Acceptable.

Degree of Responsiveness: Responsive.

Privacy and Security Protections for Patients: Acceptable.

Data Management Plan:
Acceptable.

CRITIQUE 2

Significance:	2
Investigator(s):	2
Innovation:	1
Approach:	3
Environment:	1

Overall Impact: Children with special health care needs require a significant amount of care coordination which is often left to families to accomplish, given the fragmented nature of health systems and providers. A personal health record (PHR) that captured family or patient reported outcomes that could communicate bidirectionally with the EHR would be a significant step forward for both families and providers.

To address this issue, the investigators have developed a PHR prototype which will integrate with the EHR through FHIR technology. In their study, they will evaluate the feasibility of use of a PHR and qualitatively assess the facilitators and barriers to implementation and use. Overall enthusiasm for this proposal is high, given the significant impact a PHR would have to both children and adults with special health care needs and MCC, as well as the experience of the team and institution with use of FHIR technology. The primary weakness of the proposal is that it relies on FHIR technology, which is not universally implemented and available, potentially limiting generalizability of the findings.

1. Significance:
Strengths
- 20% of children have special health care needs and require care coordination between multiple subspecialists or team.
- Communication of family health insights at home, and potentially care across disparate systems, is not easily shareable or visible to providers.
- Significant care coordination needs apply to adults with MCC as well as children with special care needs, so success would potentially impact other patient groups.

Weaknesses
- Success requires FHIR technology which has been variably utilized by health systems, thus may limit generalizability.

2. Investigator(s):
Strengths
- Team competed and won Maternal Child Health Bureau Grand Challenge on care coordination with development of their app.
- Team includes expertise in digital health, care coordination, implementation science, and mixed methods/qualitative assessment- requisite expertise is represented.
- PI runs a complex care service focused on the study population and has training in implementation science; he is devoting 20% time to application; currently holds an active K12 award.
- LOS from Duke Technology Digital Strategy Office promoting ability to integrate with FHIR standards and Boston IT who developed the app for ongoing integration.

Weaknesses
- **K**12 effort will have to be adjusted for R21.
- Very little effort support for Co-PIs (1-5%).

3. Innovation:
Strengths
- **T**he use of a personal health record that integrates with the EHR through FHIR technology to allow bidirectional sharing of information is transformative.
- A PHR prototype has been developed and assessed by families with high acceptance.
- If successful, it could be used by adults with MCC and many others to share healthcare information with various providers.
- Some risk due to FHIR technology but high reward.

Weaknesses
- Availability of FHIR technology and acceptance – not universal.
- Issues with FHIR technology – data matching, existing domain information, etc.

4. Approach:
Strengths
- Concrete use of several frameworks for both the design of the HIT application and for assessment (TAM and CFIR).
- Assessment of technical and implementation feasibility using some standard measures and also assessment of usability.
- Bidirectional communication between the EHR and app to facilitate communication of information from provider to family and from family to provider.
- Family shared information is integrated into a provider dashboard that is visible in the EHR, so does not require multiple sites for access – shows ePRO trends as well as recent visits, goals, medications.

- Evaluating meaningful outcomes that include both family perception of care (PICS for care coordination and QOL assessment) and usability as well as healthcare utilization (ED use, hospitalizations).

Weaknesses
- Not a lot of information about qualitative interviews post-implementation (themes and coding) except guidance by CFIR.
- Not clear that standardized pediatric ePROs will be utilized for family reported data (like PROMIS pediatric measures).
- Not clear the information that the provider will be evaluating and the time involved.
- 6 month follow up time period is short but necessary within the time limits of the grant.

5. Environment:
Strengths
- Both Duke and Boston have adequate resources and support to accomplish the aims of the grant.
- Duke Health Digital Strategy Office and Duke Mobile App Gateway resources.
- Duke Implementation Science Core.
- Boston Digital Health and Innovation Accelerator.
- LOS demonstrating the collaboration of the Duke DSO with Boston Digital Health and Innovation Accelerator.
- Overall description of Duke Primary Care is provided and demonstrates adequate patient numbers and distribution for study.

Weaknesses
- None noted.

Appendix: Acceptable.
SDRD is included and describes the hardware/software/security required for use of the FHIR-enabled mobile app

Budget and Period of Support: Budget is appropriate.

Protections for Human Subjects: Acceptable.
Protections appear appropriate, including for use of mobile app with PHI

Data and Safety Monitoring Plan: Acceptable.
No DSMB but adequate oversight by PI for 40 enrolled families

Single IRB for Cooperative Research: Acceptable.
Single IRB

Inclusion of Women: Acceptable.
Anticipated to have a high number of women based on past studies but appropriately justified.

Inclusion of Minorities: Acceptable.
Distribution of minorities proportional to population and clinic population.

Inclusion of Individuals Across the Lifespan: Acceptable.
Study will enroll adult parents of children with health care needs, appropriate justification provided for not enrolling children

Inclusion of Priority Populations: Acceptable.
Study targets families of children with special health care needs.

Degree of Responsiveness: Responsive.

Privacy and Security Protections for Patients: Appropriate protections in place for patients/families.

Data Management Plan: Acceptable.
Well described data management plan.

CRITIQUE 3

Significance:	3
Investigator(s):	3
Innovation:	3
Approach:	3
Environment:	2

Overall Impact: Strong scientific premise for study, study team building on significant track record of work that logically leads to this study. Study findings will provide key insights that will be critical for future scaling in an understudied but important area - use of mHealth for bidirectional communication between parents of children with special health care needs and their provider team. Slight concern re: how parent data collected will be communicated back to provider in a manner that is not disruptive to workflow (requiring a separate login account). Intervention development and assessment plan grounded in theory. Approach well-reasoned, plan well-outlined, and outcomes of interest clearly detailed. PI is somewhat junior, with limited prior experience leading NIH-funded studies.

1. Significance:
Strengths
- Addresses important issue of care coordination for families of children with special health care needs.
- Strong scientific premise for study.
- Findings would support future larger study involving multiple sites to examine effectiveness of the intervention.
- Establishing feasibility of this approach opens the door for application of this technology to other contexts.
- Bidirectional data flow - including flow easily from the EHR into patient/family-facing app.
- Supports patient-centered care delivery as this facilitates communication between families and providers.

Weaknesses
- Could focus more on how the app will address families from lower SES backgrounds, who face literacy challenges, who could particularly benefit from care coordination.
- More information would be helpful regarding the degree to which intervention will fit into the clinical workflow; appendix suggests that there may be a requirement for a separate login account for provider care team to access their dashboard.

2. Investigator(s):
Strengths
- Relatively junior PI, who is currently funded as a K12 scholar.

- Team with multi-year history of collaboration on development of the CareMap tool over past 4 years, with CareMap as a tool recognized by MCHB
- Team members with complementary expertise; well-delineated areas of expertise in Study Team Structure document.
- PI with adequate time budgeted for project.
- Letters of support included for collaborating stakeholder organizations.

Weaknesses

- Limited prior experience of PI as a PI of NIH-funded grants.
- Very limited PI effort for co-I from Boston (1%), who co-developed the app, raising some concern re: level of commitment / collaboration of the Boston site.
- Could benefit from team member with greater expertise in design of health literacy-sensitive interventions.

3. Innovation:
Strengths

- First FHIR-enabled digital personal health record mobile app to improve care coordination with secure access and sharing of EHR data and family reported health insights - not yet been tested for regular clinical care.
- Seeks to facilitate bi-directional information transfer between families of children with special health care needs.

Weaknesses

- More information would be helpful regarding the degree to which intervention will fit into the clinical workflow; appendix suggests that there may be a requirement for a separate login account for provider care team to access their dashboard.
- Unclear degree to which this app addresses or will be tailored to families facing literacy barriers.

4. Approach:
Strengths

- Focus on feasibility, will follow a group of parents/caregivers as they use the tool over 6 months, with exploration of association between higher app use and patient reported outcomes.
- Mixed methods approach a strength; qualitative approach will be important for getting detailed feedback from both parents/patients and providers.
- Outcomes include technical feasibility of having appropriate data transferred and family-reported outcomes collected, as well as implementation outcomes related to acceptability / feasibility / adoption.
- Intervention components grounded in care delivery frameworks.
- Assessment frameworks grounded in theory (TAM, CFIR).
- Will leverage EHR model that the team has developed to identify high risk children with special health care needs to be recruited into the study.
- Good discussion of potential problems/ alternative strategies.
- Well described recruitment approach.

Weaknesses

- Methods could be better described, including more details needed re: qualitative interviews.
- Limited sample size of families and providers involved - 10-12 families, 4 providers.
- Only English speaking parents to be included; would be beneficial if app could be tested for both English and Spanish-speakers.
- More preliminary data on MyChart use would be helpful, especially for disadvantaged, low literacy populations most at risk.

- For analyses exploring associations between mobile app adoption and family-reported outcomes -would be good to explore issues of whether differences in literacy level, English proficiency of families impacts adoption and moderates associations.
- Some concerns regarding patient-facing app screens - -to ensure use of plain language. Eg. terms like “insights” “asplenia”, “medication adherence” “pain crisis”

5. Environment:
Strengths
- Scientific environment will contribute to probability of success.
- Strong institutional support.
- Letters of support included from relevant partners.

Weaknesses
- Limited budgetary support designated for Boston Children’s collaborators who helped co-develop app.

Appendix: Acceptable.

Budget and Period of Support: Overall proposed budget reasonable - although low effort for Boston co-I.

Protections for Human Subjects: Acceptable.

Data and Safety Monitoring Plan: Acceptable.

Single IRB for Cooperative Research: Acceptable.

Inclusion of Women: Acceptable.

Inclusion of Minorities: Acceptable.

Inclusion of Individuals Across the Lifespan:
Acceptable.

Inclusion of Priority Populations: Acceptable.

Degree of Responsiveness: Responsive.

Privacy and Security Protections for Patient: Acceptable.
No concerns

Data Management Plan: Acceptable.

THE FOLLOWING RESUME SECTIONS WERE PREPARED BY THE SCIENTIFIC REVIEW ADMINISTRATOR TO SUMMARIZE THE OUTCOME OF DISCUSSIONS OF THE REVIEW COMMITTEE ON THE FOLLOWING ISSUES:

PROTECTION OF HUMAN SUBJECTS (Resume): ACCEPTABLE.

INCLUSION OF WOMEN PLAN (Resume): ACCEPTABLE.

INCLUSION OF MINORITIES PLAN (Resume): ACCEPTABLE.

INCLUSION OF AHRQ PRIORITY POPULATIONS PLAN (Resume): ACCEPTABLE.

COMMITTEE BUDGET RECOMMENDATIONS: The budget was recommended as requested.

Footnotes for 1 R21 HS028699-01; PI Name: Ming, David Y

+ Derived from the range of percentile values calculated for the study section that reviewed this application.

MEETING ROSTER

Healthcare Information Technology Research
AGENCY FOR HEALTHCARE RESEARCH AND QUALITY
HITR
06/03/2021 - 06/04/2021

CHAIRPERSON(S)

JENDERS, ROBERT ALLEN, BS, MD, MS
PROFESSOR OF MEDICINE AND ASSOCIATE DIRECTOR
CLINICAL AND TRANSLATIONAL SCIENCE INSTITUTE
UNIVERSITY OF CALIFORNIA, LOS ANGELES
LOS ANGELES, CA 90059

MEMBERS

ASAN, ONUR, PHD
ASSOCIATE PROFESSOR
SCHOOL OF SYSTEMS AND ENTERPRISES
STEVENS INSTITUTE OF TECHNOLOGY, NEW JERSEY
HOBOKEN, NJ 07030

ASCHE, CARL, PHD, MSC, MS
PROFESSOR
DEPARTMENT OF MEDICINE
UNIVERSITY OF ILLINOIS
ONE ILLINI DRIVE
PEORIA, IL 61605

CANTOR, MICHAEL, MD, MA
EXECUTIVE DIRECTOR
CLINICAL INFORMATICS
REGENERON GENETICS CENTER
TARRYTOWN, NY 10591

CARON, ALEECE, PHD
ASSOCIATE PROFESSOR OF MEDICINE
CENTER FOR HEALTHCARE, RESEARCH, AND POLICY
THE METROHEALTH MEDICAL CENTER
CASE WESTERN RESERVE UNIVERSITY
CLEVELAND, OH 44109

CHUI, MICHELLE ANNE, PHD, PHMD
HAMMEL SANDERS PROFESSOR
SOCIAL & ADMINISTRATIVE SCIENCES
SCHOOL OF PHARMACY
UNIVERSITY OF WISCONSIN
MADISON, WI 53705

KOSZALINSKI, REBECCA SUSAN, PHD, MS, BSN
ASSOCIATE PROFESSOR
OUHSC CON
UNIVERSITY OF OKLAHOMA
OKLAHOMA CITY, OK 73117

LANHAM, HOLLY J., PHD, MBA
ASSOCIATE PROFESSOR
DEPARTMENT OF MEDICINE
DIVISION OF GENERAL AND HOSPITAL MEDICINE
UNIVERSITY OF TEXAS HEALTH SAN ANTONIO
SAN ANTONIO, TX 78229

LEROY, GONDY, PHD
ELLER FELLOW AND ASSOCIATE PROFESSOR OF MIS,
PRESIDENT OF AIS SIG-HEALTH
MANAGEMENT INFORMATION SYSTEMS
ELLER COLLEGE OF MANAGEMENT
UNIVERSITY OF ARIZONA
TUCSON, AZ 85721

LUCERO, ROBERT J, MSN, PHD, BSN, MPH *
ASSOCIATE DEAN FOR DIVERSITY, EQUITY AND INCLUSION
UCLA SCHOOL OF NURSING
700 TIVERTON AVENUE, ROOM 4-266
LOS ANGELES, CA 90095

MELTON, BRITTANY LEE, PHD, PHMD
ASSOCIATE PROFESSOR
DEPARTMENT OF PHARMACY PRACTICE
UNIVERSITY OF KANSAS
KANSAS CITY, KS 66160

MELTON, KRISTIN R, MD
ASSOCIATE PROFESSOR OF PEDIATRICS
DIVISION OF NEONATOLOGY, PULMONARY AND
PERINATAL BIOLOGY
UNIVERSITY OF CINCINNATI
CINCINNATI, OH 45229

MOSS, JACQUELINE A, PHD, FAAN, RN
PROFESSOR AND ASSOCIATE DEAN FOR TECHNOLOGY &
INNOVATION
SCHOOL OF NURSING
UNIVERSITY OF ALABAMA AT BIRMINGHAM
HOOVER, AL 35244

PACE, WILSON DOUGLAS, MD *
PROFESSOR EMERITUS OF FAMILY MEDICINE
UNIVERSITY OF COLORADO
SCHOOL OF MEDICINE
CHIEF MEDICAL OFFICER
DARTNET INSTITUTE
AURORA, CO 80045

PAYNE, THOMAS H., MD *
PROFESSOR OF MEDICINE, BIOINFORMATICS AND MEDICAL EDUCATION
UNIVERSITY OF WASHINGTON SCHOOL OF MEDCINE
AND SCHOOL OF PUBLIC HEALTH
SEATTLE, WA 98195

RAO, GOUTHAM, MD
PROFESSOR AND CHAIRMAN
FAMILY MEDICINE & COMMUNITY HEALTH
CASE WESTERN RESERVE UNIVERSITY &
UNIVERSITY HOSPITALS OF CLEVELAND
CLEVELAND, OH 44106

ROSSETTI, SARAH COLLINS, PHD, RN
ASSISTANT PROFESSOR OF BIOMEDICAL INFORMATICS AND NURSING
DEPARTMENT OF BIOMEDICAL INFORMATICS
SCHOOL OF NURSING
COLUMBIA UNIVERSITY MEDICAL CENTER
NEW YORK, NY 10032

SCHULMAN, DANIEL JASON, BS, PHD *
SENIOR SCIENTIST
ACUTE CARE SOLUTIONS
PHILIPS RESEARCH NORTH AMERICA
CAMBRIDGE, MA 02141

SORONDO, BARBARA, MBA, MD
DIRECTOR
CLINICAL DEVELOPMENT
LG CHEM LIFE SCIENCES
INNOVATION CENTER, INC.
CAMBRIDGE, MA 02142

TABER, DAVID J., PHMD, MS
PROFESSOR
DEPARTMENT OF SURGERY
DIVISION OF TRANSPLANT SURGERY
MEDICAL UNIVERSITY OF SOUTH CAROLINA
CHARLESTON, SC 29425

WELLS, BRIAN JAY, MD, PHD
ASSOCIATE PROFESSOR
DEPARTMENT OF BIOSTATISTICAL AND DATA SCIENCE
WAKE FOREST SCHOOL OF MEDICINE
MEDICAL CENTER BOULEVARD
WINSTON-SALEM, NC 27157

XIE, ANPING, PHD
ASSISTANT PROFESSOR
ARMSTRONG INSTITUTE FOR PATIENT SAFETY
DEPT. OF ANESTHESIOLOGY & CRITICAL CARE MEDICINE
JOHNS HOPKINS SCHOOL OF MEDICINE
BALTIMORE, MD 21202

YIN, HSIANG, MD
ASSOCIATE PROFESSOR OF PEDIATRICS
NYU SCHOOL OF MEDICINE
550 FIRST AVENUE, NBV8E14
NEW YORK, NY 10016

ZHENG, KAI, PHD
ASSOCIATE PROFESSOR
DEPARTMENT OF INFORMATICS
DONALD BREN SCHOOL OF INFO. AND COMPUTER SCIENCES
UNIVERSITY OF CALIFORNIA, IRVINE
IRVINE, CA 92697-3440

ZHU, XI, PHD
ASSOCIATE PROFESSOR
DEPARTMENT OF HELATH MANAGEMENT AND POLICY
THE UNIVERSITY OF IOWA
COLLEGE OF PUBLIC HEALTH
145 N. RIVERSIDE DR. N222 CPHB
IOWA CITY, IA 52242

SCIENTIFIC REVIEW OFFICER

APONTE, BORIS, PHD
DIVISION OF SCIENTIFIC REVIEW
OFFICE OF EXTRAMURAL RESEARCH, EDUCATION
AND PRIORITY POPULATIONS
AGENCY FOR HEALTHCARE RESEARCH AND QUALITY
ROCKVILLE, MD 20857

EXTRAMURAL SUPPORT ASSISTANT

GAINES, CHRISTOPHER M, MBA
PROGRAM ANALYST
DIVISION OF SCIENTIFIC REVIEW
OFFICE OF EXTRAMURAL RESEARCH, EDUCATION &
PRIORITY POPULATIONS
AGENCY FOR HEALTHCARE RESEARCH & QUALITY
ROCKVILLE, MD 20850

* Temporary Member. For grant applications, temporary members may participate in the entire meeting or may review only selected applications as needed.

Consultants are required to absent themselves from the room during the review of any application if their presence would constitute or appear to constitute a conflict of interest.
